# Efficacy of posterior capsular release for flexion contracture in posterior-stabilized total knee arthroplasty

**DOI:** 10.1186/s40634-021-00422-2

**Published:** 2021-11-04

**Authors:** Tomofumi Kinoshita, Kazunori Hino, Tatsuhiko Kutsuna, Kunihiko Watamori, Takashi Tsuda, Hiromasa Miura

**Affiliations:** Department of Orthopedic Surgery, Ehime University Graduate School of Medicine, Shitsukawa, Toon, Ehime 791-0295 Japan

**Keywords:** Posterior capsular release, Osteoarthritis, Flexion contracture, Knee extension angle, Cadaveric study, Cadaveric knee, Navigation system, Total knee arthroplasty

## Abstract

**Purpose:**

Posterior capsular contracture causes stiffness during knee extension in knee osteoarthritis. Furthermore, in posterior-stabilized total knee arthroplasty (PS-TKA), a unique design such as the cam mechanism could conflict with the posterior capsule (PC) causing flexion contracture (FC). However, few studies have focused on the anatomical aspects of the PC. This study aimed to investigate the anatomical site and forms of posterior capsular attachment to the femoral cortex, and to evaluate the efficacy of posterior capsular release for FC by assessing changes in knee extension angles using a navigation system.

**Methods:**

Attachment sites of the PC were investigated in 10 cadaveric knees using computed tomography. PS-TKA was performed in six cadaveric knees using a navigation system to evaluate the efficacy of posterior capsular release for FC. Posterior capsular release was performed stepwise at each part of the femoral condyle.

**Results:**

The gastrocnemius tendon and PC were integrally attached to the femoral cortex at the medial and lateral condyles, whereas the PC at the intercondylar fossa was independently attached directly to the femoral cortex. Moreover, the PC at the intercondylar fossa was attached most distally among each femoral condyle. Posterior capsular release at the intercondylar fossa allowed 11.4° ± 2.8° improvement in knee extension. This angle was further improved by 5.5° ± 1.3°, after subsequent capsular release at the medial and lateral condyles.

**Conclusion:**

The forms and sites of posterior capsular attachment differed based on the part of the femoral condyle. Stepwise posterior capsular release was effective for FC in PS-TKA.

**Level of evidence:**

III.

## Background

Total knee arthroplasty (TKA) aims to relieve severe pain and modify limb alignment to improve patients’ quality of life. Despite the development of modern TKA procedures, approximately 20% of patients are unsatisfied with their postoperative outcomes [3]. Among the factors affecting patient satisfaction, postoperative range of motion has an obvious influence on clinical results [14]. In particular, residual flexion contracture results in discomfort and difficulty in patients’ daily life, causing clinical symptoms [5, 7, 10, 22]. In osteoarthritic knees, multiple elements could account for flexion contracture. Bone impingement with osteophytes causes severe flexion contracture [25]. Moreover, soft-tissue adhesions caused by trauma or infection influence the range of motion of the knee joint. Additionally, posterior capsular contracture is the leading cause of flexion contracture. In TKA, deficiencies in surgical techniques for managing posterior capsular contracture could lead to poor clinical outcomes. Particularly, in posterior-stabilized TKA (PS-TKA) that has a unique design such as the cam mechanism, the posterior capsule (PC) could conflict with the post-cam mechanism at the intercondylar fossa, resulting in flexion contracture.

Intraoperative flexion contracture occurs even with accurate bone resection and optimal management of soft tissues as planned preoperatively. Hence, many studies have explored appropriate surgical techniques for its management [1, 18]. The intraoperative extension gap is related to the postoperative knee extension angle [18]. Besides, previous studies have demonstrated the efficacy of additional distal femoral bone cutting to improve the extension gap and intraoperative knee extension angle [2, 11]. Additionally, posterior clearance affects the postoperative knee extension angle. To achieve adequate posterior clearance, osteophyte removal and posterior capsular release are widely known to be critical surgical methods [24, 29]. To improve flexion contracture, surgeons should employ additional surgical procedures. However, there is no consensus on the type of adjunctive surgical procedure preferred to improve flexion contracture with an optimal soft tissue balance. Furthermore, in cases with severe flexion contracture, surgeons should consider using a combination of several surgical procedures. Therefore, knowledge of the actual efficacy of such surgical techniques is crucial for the optimal selection of these technique, intraoperatively. However, little attention has been paid to the actual efficacy of posterior capsular release for knee extension angle in comparison with other surgical methods to improve flexion contracture. Furthermore, few studies have researched the anatomical aspect of the posterior side of the knee joint [17, 19]. A better understanding of the anatomical form and sites of the PC of the knee joint would facilitate posterior capsular release.

This cadaveric study aimed (1) to investigate the anatomical attachment site and form of the PC of a knee joint to the femoral cortex and (2) to evaluate the efficacy of posterior capsular release for flexion contracture by measuring changes in the knee extension angle using a navigation system. We hypothesized that the attachment site and form of the PC differed depending on the part of the femoral condyle, and that the efficacy of posterior capsular release also varied depending on the released part of the femoral condyle.

## Methods

**Fig. 1:**
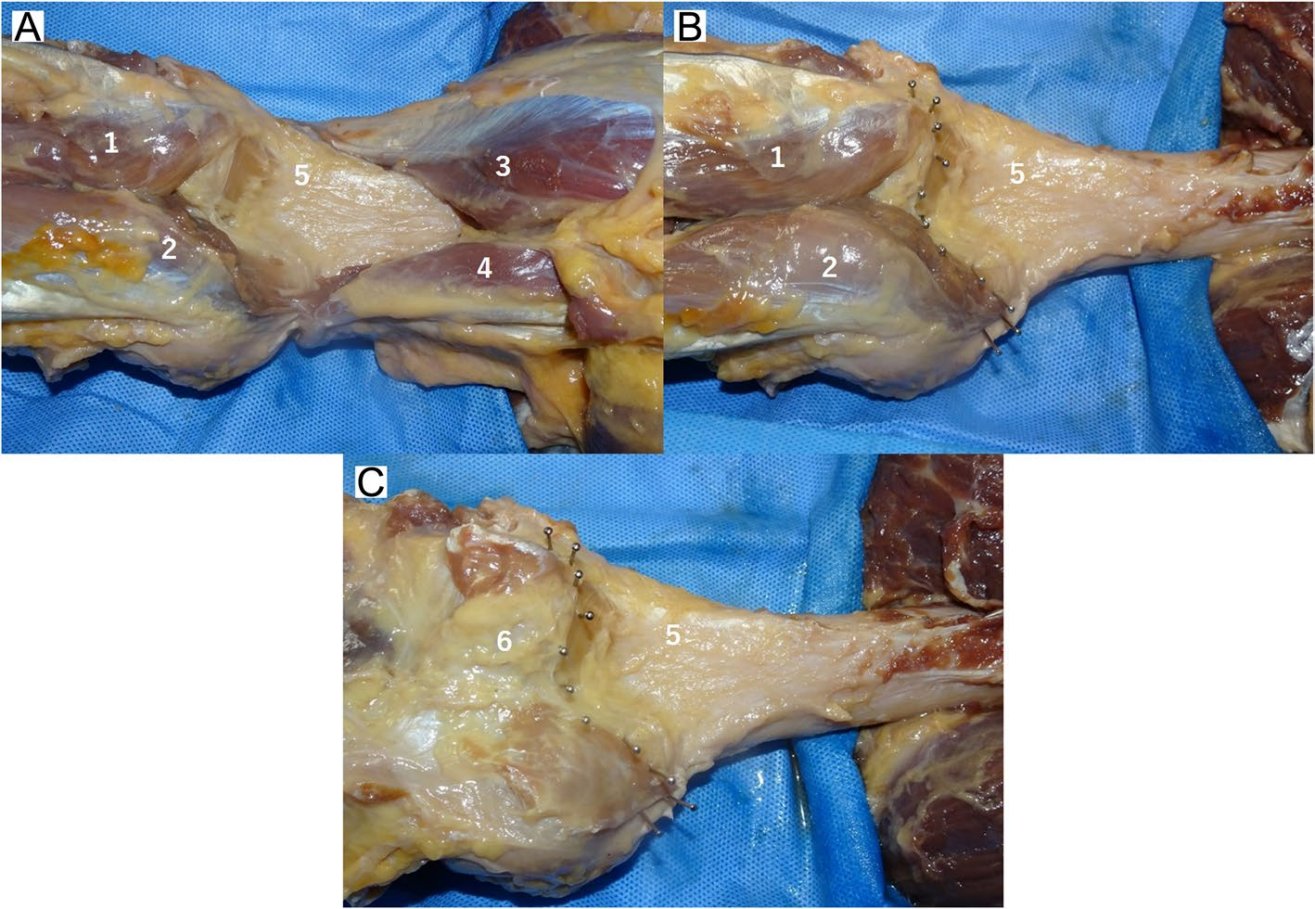
Posterior aspect of a knee joint. **A** Posterior view of the femur; **B** Posterior view of the femur, showing the location of the femoral attachment of the posterior capsule of the knee joint with stainless pins; **C** After excision of the gastrocnemius muscle. 1: lateral head of the gastrocnemius tendon; 2: medial head of the gastrocnemius tendon: 3: biceps femoris muscle; 4: semimembraneous muscle; 5: posterior aspect of the femur; 6: posterior capsule of the knee joint

**Fig. 2:**
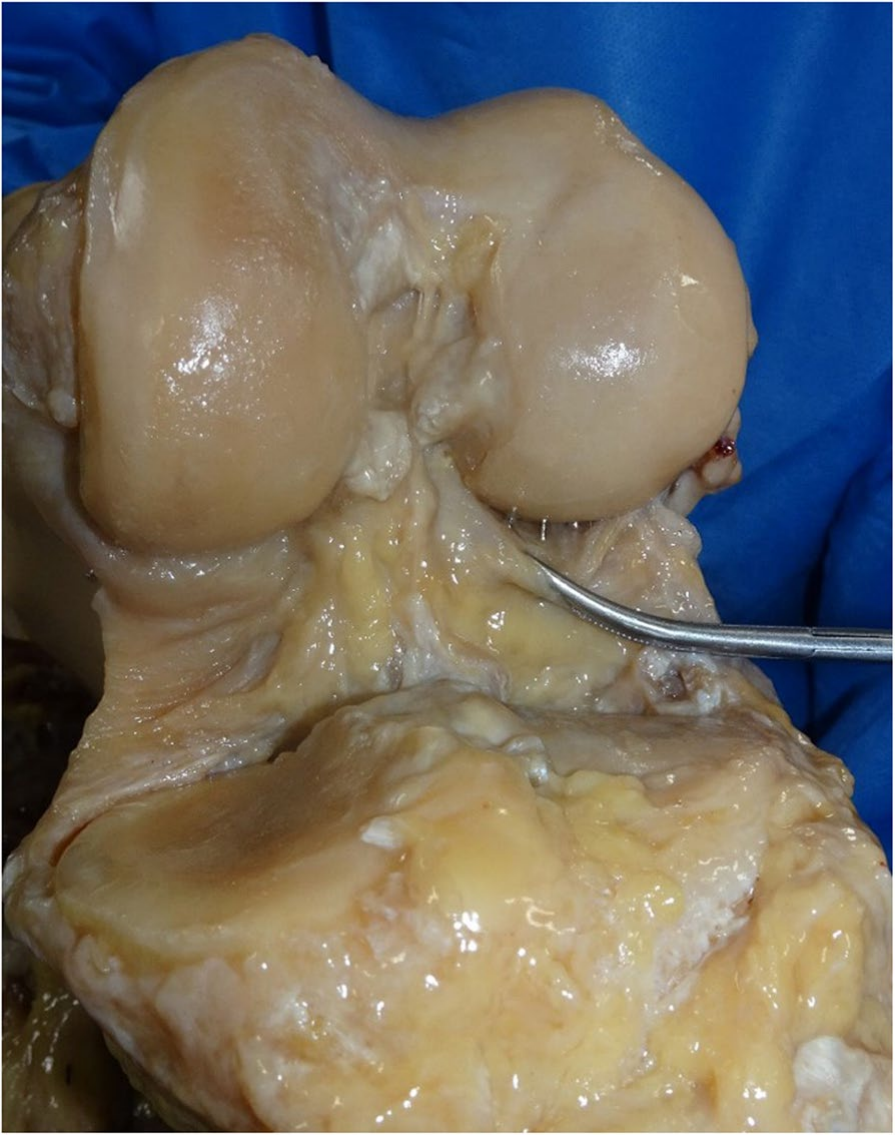
The attachment site of posterior capsule observing from the anterior side of the knee joint

**Fig. 3:**
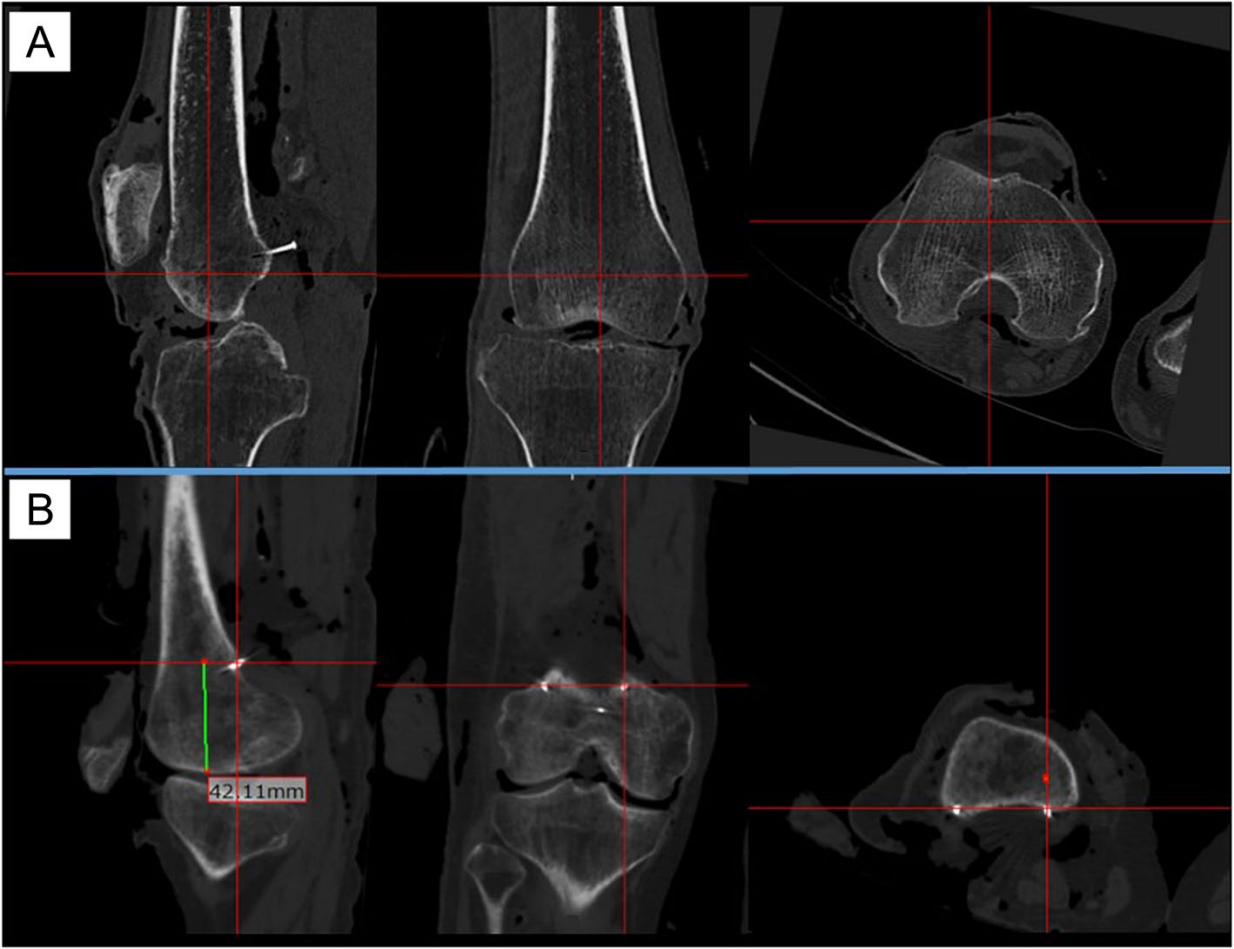
Evaluation of the attachment site of the posterior capsule. **A** Coronal, sagittal, and axial plane computed tomography images evaluating the attachment site of the posterior capsule at each condyle of a femur; **B** Evaluation of the attachment site of the posterior capsule at the medial condyle. The red cross lines in this figure focus on the attachment site of the posterior capsule marked with a stainless pin

**Fig. 4:**
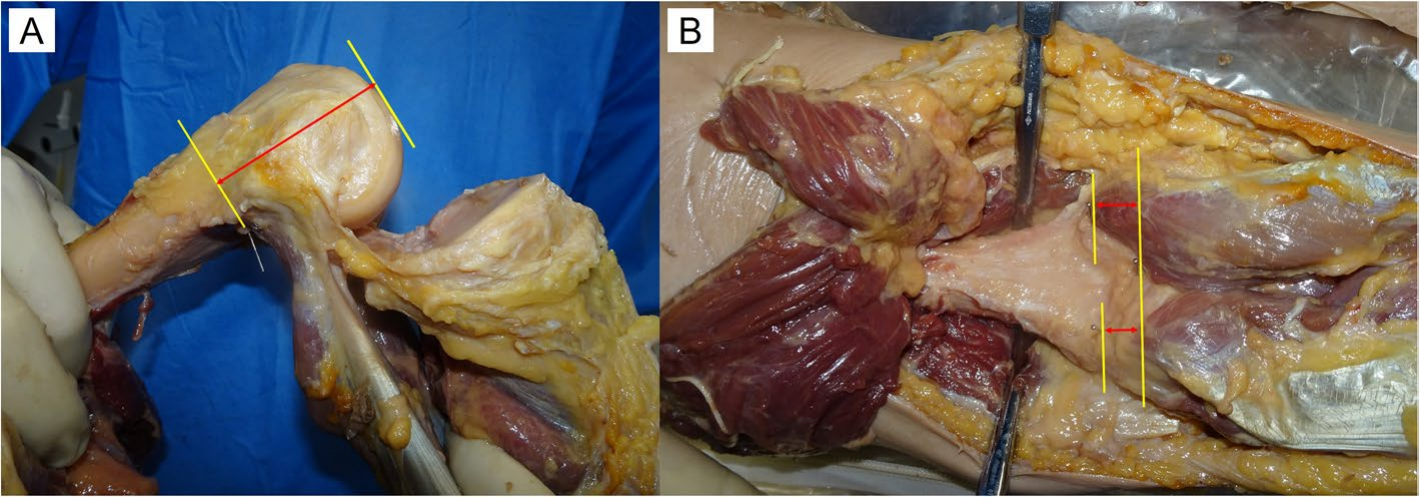
Each measurement method. **A** The length from the most proximal attachment sites of the posterior capsule to the distal femoral point at each condyle; **B** The length between the most proximal attachment site of the posterior capsule at intercondylar fossa and the most proximal attachment site of the posterior capsule at each condyle

In this study, 10 knees of five cadavers (four male and one female) under Thiel fixation were examined to evaluate the anatomical aspect of the PC [27]. The mean patient age at the time of death was 79.2 ± 5.2 (range, 69–83) years. None of the cadavers had any history of knee surgery and any macroscopic degenerative or traumatic changes. Preparation began with the removal of all extra-articular soft tissues by dissection using a posterior approach. Initially, a midline straight incision that was 15 cm proximal to and 15 cm distal from the joint line was made on the posterior side of the knee joint. The subcutaneous tissue was opened to expose the posterior knee muscle. The semitendinosus muscle was subsequently dissected, and the gastrocnemius and plantar muscles attached to the posterior femoral cortex were identified. After dissection of the posterior muscles, stainless steel pins were subsequently inserted at the site of the PC attachment at the medial condyle, lateral condyle, and intercondylar fossa (Figs. 1, 2). Computed tomography (CT) was subsequently performed to measure the site of the attachment of the PC. In this study, we evaluated the precise attachment site of the PC using a three-dimensional planning and evaluation tool (Zed Knee, LEXI Co., Ltd., Tokyo, Japan). The axis of the coronal and sagittal view was defined as the directions parallel to the femoral bone axis, and an axis of the axial view was set parallel with the Whiteside line (Fig. 3). The anatomical characteristics of the 10 cadaveric knees, measured using CT, are presented in Table 1. We measured the length between the most proximal attachment sites of the PC to the distal femoral points in the axis of the sagittal view at each condyle using CT (Fig. 4). Additionally, we measured the length between the attachment site of the PC at intercondylar fossa to the attachment site of the PC at each condyle in the sagittal view using CT (Fig. 4). All the data were collected by the same surgeon, and the test–retest reliability for evaluating the length using ZedKnee indicated that the interclass and intraclass correlation coefficients were sufficiently high, with values of > 0.9.

**Table 1:** Anatomical characteristics of 10 cadaveric knees

		Total
		(Mean ± SD)
MC depth	(mm)	30.1 ± 3.2
LC depth	(mm)	24.0 ± 2.8
Medial PCO length	(mm)	63.6 ± 5.5
Lateral PCO length	(mm)	63.2 ± 7.4
Bicondylar width	(mm)	82.3 ± 7.7

*MC depth* maximum anteroposterior diameter of the medial femoral condyle, *LC depth* maximum anteroposterior diameter of the lateral femoral condyle, *medial PCO length* medial posterior condylar offset length, *lateral PCO length* lateral posterior condylar offset length, *bicondylar width* length between the medial and lateral epicondyles, *SD* standard deviation

**Fig. 5:**
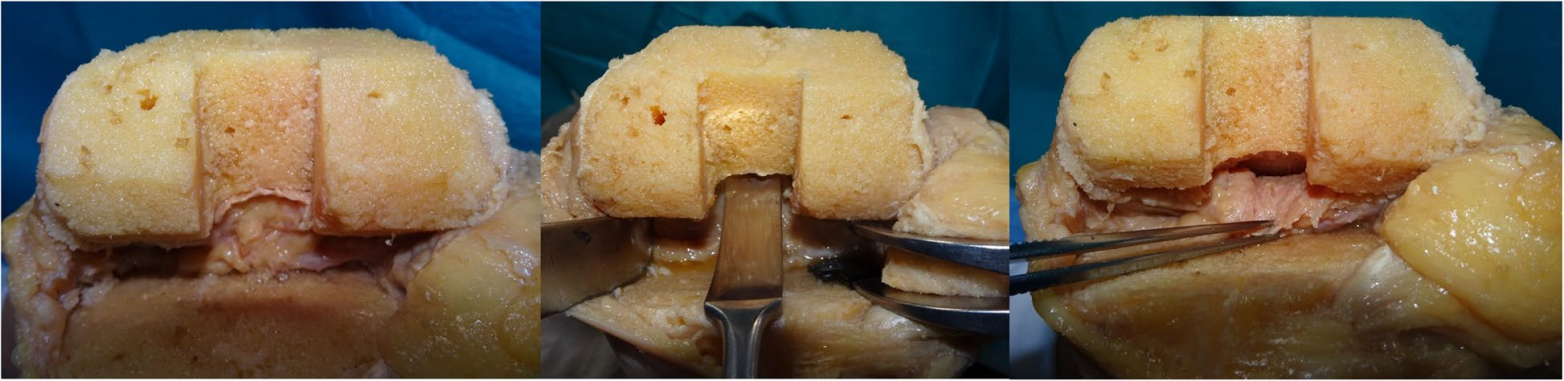
Posterior capsular release using a curved osteotome. The left figure shows the attachment site of the posterior capsule at the intercondylar fossa of a femur. The middle figure shows posterior capsular release at the intercondylar fossa. The posterior capsule was completely detached from the posterior femoral cortex. A spreader was used to create adequate space to perform posterior capsular release. The right figure shows the attachment site of the posterior capsule at the intercondylar fossa after capsular release

To evaluate the efficacy of the posterior capsular release, six cadaveric knees with no history of knee surgery and no traumatic changes were analyzed. PS-TKA (Persona PS: Zimmer, Warsaw, Indiana, USA) was performed using a measured resection technique with a navigation system (Precision Knee Navigation Software version 4.0, Stryker, Kalamazoo, Michigan, USA). All the patients had medial knee osteoarthritis. The study population comprised five male knees with a mean age of 83.2 ± 5.1 (range, 79–95) years. First, specific anatomical reference points were located by anchoring infrared signal transducers into the femur and tibia with pins. A skin incision was made to expose the subcutaneous tissue. Registration was performed with osteophytes and soft tissues, including anterior cruciate ligament preservation. The anteroposterior and rotational axes of the femur and tibia were identified with respect to the femoral and tibial anatomical landmarks. Next, the distal femur was cut using a navigation system with a measured resection technique. The proximal tibial cut was made perpendicular to the mechanical axis of the tibia, based on the navigation system, confirming a 10-mm-thick proximal lateral tibial bone resection and a 3° tibial slope. After removing the posterior condylar osteophytes, the trial components with a 10-mm trial insert were placed and the joint capsule was temporarily closed using four suture strands. Thereafter, the knee extension angle was measured using the navigation system. Posterior capsular release was subsequently performed at the intercondylar fossa using a curved osteotome (Fig. 5), and the knee extension angle was measured with the same trial components using the navigation system. The same procedures were repeated at the lateral condyle and medial condyle. A single surgeon performed all the procedures using a curved osteotome. The change in the knee extension angle was calculated following the posterior capsular release at each attachment site. The PC was released in the order of the intercondylar fossa, lateral condyle, and medial condyle. The PC attached to the intercondylar fossa was completely released from the femoral cortex. At the medial and lateral condyles, the PC was released completely with the width of the bone resection of the posterior condyle.

Ethical approval for this study was obtained from the ethical committee of our institution and written informed consent was obtained from all patients prior to their demise.

### Statistical analysis

Non-parametric Wilcoxon’s signed-rank tests were performed to identify the difference between the extension angles at each time point. Analyses were performed using JMP version 14.0 (SAS Institute, Tokyo, Japan). *P*- values< 0.05 were considered statistically significant.

## Results

### The anatomical site and forms of attachment of the posterior capsule of a knee joint

**Fig. 6:**
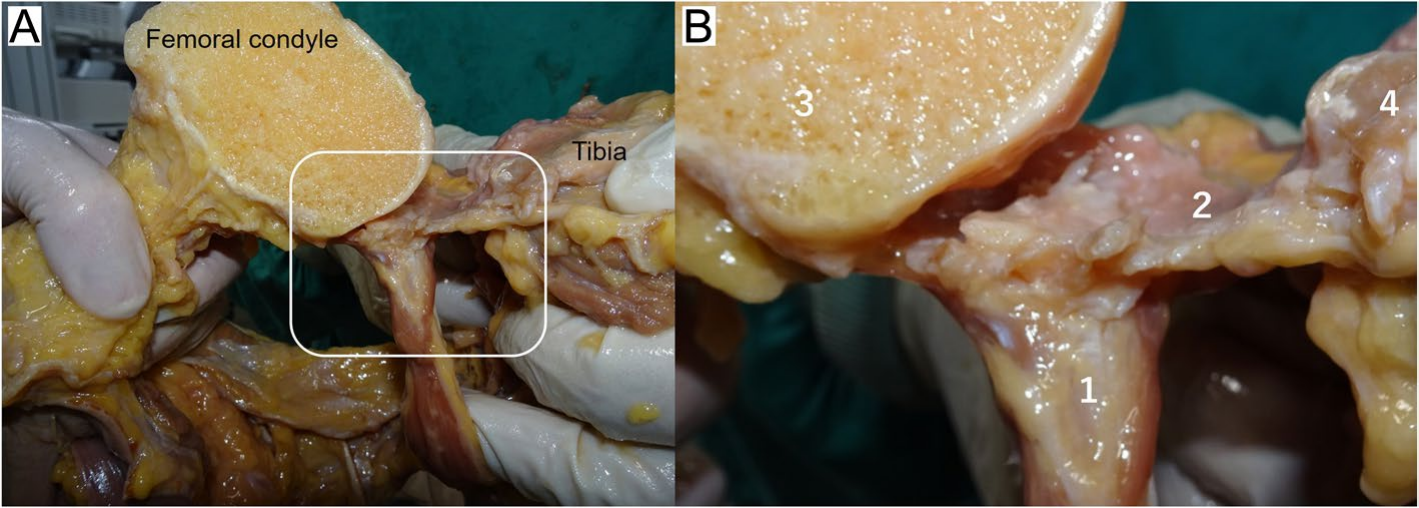
The attachment form of the posterior capsule of a knee joint to the femoral cortex. **A** Cross-section of the medial condyle of a femur. The white square shows the attachment site of the gastrocnemius tendon and the posterior capsule to the femoral cortex. The gastrocnemius tendon and posterior capsule were attached integrally to the femoral cortex at the medial condyle; **B** An enlarged view of the attachment site. 1: medial head of the gastrocnemius tendon; 2: posterior capsule of a knee joint; 3: femoral condyle; 4: tibia

The attachments of the PC to the femoral cortex differed depending on the part of the femoral condyle involved. The gastrocnemius tendon and PC were attached integrally to the posterior cortex of the femur at the medial and lateral condyles (Fig. 6). Conversely, the PC was attached directly to the femoral cortex at the intercondylar fossa. In all cases, the PC was attached most distally at the intercondylar fossa. The PC was attached 10.3 ± 3.4 mm proximally at the medial part and 9.4 ± 3.8 mm proximally at the lateral part compared with the attachment site at the intercondylar fossa (Table 2). Table 3 shows the length between the distal femoral point and the PC attachment. Although the distal femoral point was different at each condyle, the length between the distal femoral point and the attachment site of the PC at the medial condyle was longer than the lengths at the intercondylar fossa and lateral condyle in all cases.

**Table 2:** Length between the posterior capsule attachment site at the intercondylar fossa and at each condyle

		Length from the attachment site of the PC at the intercondylar fossa to the attachment site of the PC at each condyle	
		Medial condyle (mm)	Lateral condyle (mm)
Case 1	Right	14.4	12.9
	Left	12.0	10.1
Case 2	Right	16.0	15.2
	Left	12.7	14.7
Case 3	Right	5.4	7.0
	Left	9.1	7.0
Case 4	Right	10.4	11.6
	Left	9.8	6.7
Case 5	Right	6.5	5.6
	Left	6.2	3.6
Mean ± SD		10.3 ± 3.4	9.4 ± 3.8

*PC* posterior capsule, *SD* standard deviation

**Table 3:** Length between the distal femoral point and the attachment site of the posterior capsule

	R/L	Lateral condyle (mm)	Intercondylar fossa (mm)	Medial condyle (mm)
Case 1	Right	34.2	26.5	42.1
	Left	38.3	19.4	39.9
Case 2	Right	45.5	29.5	53.2
	Left	38.9	30.8	49.6
Case 3	Right	40.1	29.7	48.5
	Left	39.3	25.6	46.9
Case 4	Right	38.8	29.1	46.9
	Left	37.9	29.5	47.3
Case 5	Right	36.6	26.4	43.0
	Left	37.0	25.4	46.2
Mean ± SD		38.7 ± 2.8	27.2 ± 3.2	46.4 ± 3.6

*SD* standard deviation

### Efficacy of posterior capsular release for flexion contracture 

**Fig. 7:**
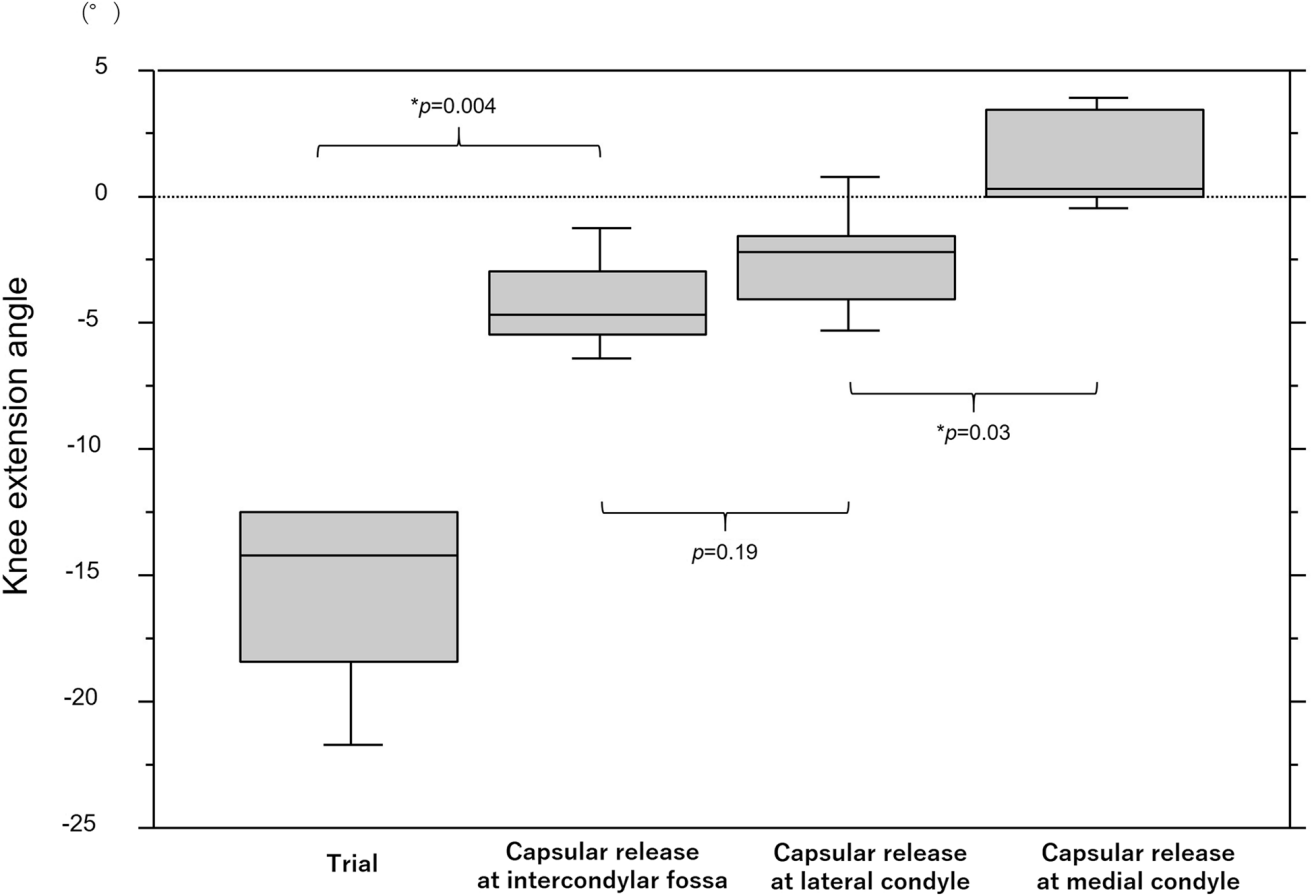
The changes in the knee extension angle following stepwise posterior capsular release. Figure shows box and whisker plot of changes in the knee extension angle after posterior capsular release at each femoral condyle. * Significant difference in the knee extension angle between before and after capsular release at each step (*p* < 0.05)

Figure 7 represents the changes in the knee extension angle following the posterior capsular release at each condyle. The mean knee extension angles were − 15.6° ± 3.5° with trial component, − 4.3° ± 1.8° following capsular release at the intercondylar fossa, − 2.4° ± 2.0° following capsular release at the lateral condyle and 1.3° ± 1.8° after capsular release at the medial condyle. The knee extension angle showed statistically significant changes following capsular release at the intercondylar fossa and at the medial condyle. The mean change in extension angle following capsular release at the intercondylar fossa was 11.4° ± 2.8°. Furthermore, the extension angle was improved by 5.5° ± 1.3° with additional capsular release at the medial and lateral condyles.

## Discussion

The most important finding of the present study was that the attachment site of the PC differed at each femoral condyle. The PC was attached most distally at the intercondylar fossa compared with other parts of the femoral condyle in all patients of our study. This result suggested that the effectiveness of posterior capsular release differed based on the part of the PC that was released. In TKA, achieving optimal posterior clearance has a significant effect on the postoperative range of motion. Posterior condylar offset increased following implantation even with optimal bone resection and achieved a rectangular gap intraoperatively, thus demonstrating a negative effect on postoperative knee flexion [6]. Moreover, increasing the posterior condylar offset reportedly influenced the component gap in knee extension [28]. Considering these findings together with those of the present study, in addition to optimal bone resection, achieving an ideal posterior clearance by PC release is conclusive. Moreover, surgeons often select larger femoral components in PS-TKA on account of the influence of posterior cruciate ligament resection to ensure optimal flexion gap. This strategy may increase posterior condyle offset after implantation. Furthermore, in PS-TKA with a unique post-cam design, adjustment using posterior capsular release at the intercondylar fossa that is attached most distally to the femoral condyle is considered a refined surgical technique that helps control the soft tissue balance intraoperatively.

In addition to the posterior capsular release, previous studies have reported on surgical methods for improving intraoperative flexion contracture [1, 2, 11, 18, 24, 29]. Osteophyte removal from the posterior femoral condyle improves the knee extension angle in TKA [11]. Moreover, additional distal femoral resection has been shown to be an efficient surgical method for improving flexion contracture [24, 29]. In contrast, joint line elevation by additional distal femoral bone resection has been a clinical concern for decades. Previous cadaveric studies have reported the relationship between additional distal femoral resection and mid-flexion instability by joint line elevation and indicated that excessive joint line elevation following TKA affected functional outcomes [13]. However, in patients with varus knee osteoarthritis, joint elevations of < 2 mm were not correlated with mid-flexion laxity [16]. The efficacy of 2-mm additional distal femoral resection for the knee extension angle was reported to be 3.6°–9° [12, 15, 24, 26, 29]. This study demonstrated an improvement of 11.4° ± 2.8° at the intercondylar fossa following posterior capsular release, showing an equivalent or better effect than that reported for the 2-mm additional bone resection. Moreover, as its advantage, posterior capsular release achieves an optimal knee extension angle, regardless of joint line elevation. Accordingly, dealing with intraoperative flexion contracture by posterior capsular release is a skill that surgeons performing TKA should learn to achieve better clinical results.

In this study, we performed posterior capsular release and confirmed complete PC detachment from the femoral cortex. At the level of the PC attachment site, the popliteal artery and vein run close to the capsule of the knee joint. The tibial nerves are located at the side and above the artery and vein. The popliteal artery courses through the fossa from the distal portion of the adductor magnus muscle and runs close to the PC of the knee joint. Thereafter, the artery enters diagonally and branches into the genicular arteries [17]. Hence, excessive posterior capsular release could damage these structures owing to their anatomical location. Sanz et al. reported that the popliteal artery and the common peroneal nerve are located 1.01 and 1.74 cm (average) posterior to the posterior horn of the lateral meniscus, respectively. In addition, they mentioned that the distance from the posterior horn to the popliteal artery and common peroneal nerve did not correlate with the patient’s height, body mass index, and tibial plateau diameter [23]. Moreover, previous studies have reported on the variations of the branches of the popliteal artery [19, 20]. They demonstrated that the posterior artery has several patterns and divides into the anterior tibial artery, posterior tibial artery, and fibular artery. The popliteal artery branches distal to the tibial plateau. To our knowledge, no study has reported anomalous branches proximal to the tibial plateau at the same level as the PC attachment. Further, Pinter et al. demonstrated the safety of posterior capsular release during TKA in their cadaveric study [21]. They mentioned that the neurovascular band has no direct contact with the femoral cortex. Based on the results of previous studies and the anatomical form of the attachment of PC, surgeons could perform capsular release safely if they maintain the direction of the osteotome to the cortex of the femur and avoid directing it to the posterior neurovascular bundle. We have already performed this procedure in numerous cases but have never encountered neurovascular injuries. Furthermore, the flexion knee position has been proven to prevent the risk of injury to the popliteal artery [4]. Despite the safety of this procedure, one must be cautious to avoid damage to the neurovascular band. Training with a senior surgeon to familiarize this procedure is necessary to ensure patient safety.

The strength of our study is the use of a navigation system, which enabled precise evaluation of the change in the knee extension angle. Accurate evaluation by recent technological advances, such as a navigation system, has been already demonstrated [8, 9, 11]. We were able to evaluate a slight change in the extension angle that was difficult to detect without a navigation system. Moreover, the same coordinates were used for the measurement of the knee extension angle using a navigation system before and after posterior capsular release. This procedure enabled us to compare the changes in the knee extension angle following posterior capsular release more accurately. In addition, we assessed the change in the knee extension angles following capsular release at each condyle. To our knowledge, no study has focused on the differences in improvements of knee extension angle among parts of the released capsule. Furthermore, our study has made it clear that the efficacy of capsular release is different among the parts of the femoral condyle. The novelty of our study is on the anatomical aspects of the PC and its clinical consequences.

This study has certain limitations. First, the evaluation method of the form of attachment of the PC was insufficient. We performed macroscopic assessment and did not evaluate the attachment using a pathological method. Second, we did not perform preoperative imaging assessment of the limb alignment and degree of decline of the cartilage of cadaveric knees that underwent TKA. Third, we were unable to evaluate the temporal change in the extension angle following capsular release, since it was a cadaveric study. Hence, further research is required to investigate the effect of posterior capsular release on the postoperative knee extension angle. Moreover, we released the attachment of the PC from the femoral cortex completely by the width of the osteotomy of the posterior condyle. Considering the form of attachment of the PC, the attachment site of the gastrocnemius tendon could be detached from the femoral cortex at the medial and lateral condyles. Although this procedure is effective for increasing the knee extension angle, further studies are required to examine the influence of posterior capsular release at the medial and lateral condyles on postoperative knee pain and muscle strength. Meanwhile, from the results of this study, capsular release at the intercondylar fossa is an appropriate and effective surgical technique for intraoperative flexion contracture.

Our results revealed that the attachment site of the PC differed based on the part of the femoral condyle involved. Considering the difference in the attachment sites, we should consider using selective posterior capsular release. To prevent the conflict between the cam mechanism and the PC of the knee, posterior capsular release at the intercondylar fossa is valid in PS-TKA. In this study, posterior capsular release at the intercondylar fossa resulted in 11.4° ± 2.8° improvements in the knee extension angle after PS-TKA. Moreover, in PS-TKA, additional posterior release at each condyle is necessary when dealing with flexion contracture due to the increasing incidence of posterior condylar offset caused by the increasing size of the femoral component. Our study also demonstrated the efficacy of additional posterior capsular release, which resulted in improvements of 5.5° ± 1.3° in knee extension. Thus, selective posterior capsular release is an effective surgical method for addressing flexion contracture in TKA. Our data comprise important implications for the surgeon’s selection of surgical method when they encounter flexion contracture intraoperatively.

## Conclusion

The form and site of attachment of the PC varied depending on the part of the femoral condyle. Stepwise posterior capsular release was effective for intraoperative flexion contracture in posterior-stabilized total knee arthroplasty.

## Abbreviations

TKA: Total knee arthroplasty
PC: Posterior capsule
FC: Flexion contracture
PS-TKA: Posterior-stabilized total knee arthroplasty
